# Supplementary figures and images for: Uncharacterized and lineage-specific accessory genes within the Proteus mirabilis pan-genome landscape
Source: mSystems. 2023 Jun 21;8(4):e00159-23. doi: 10.1128/msystems.00159-23 (PMC10469602; doi:10.1128/msystems.00159-23)

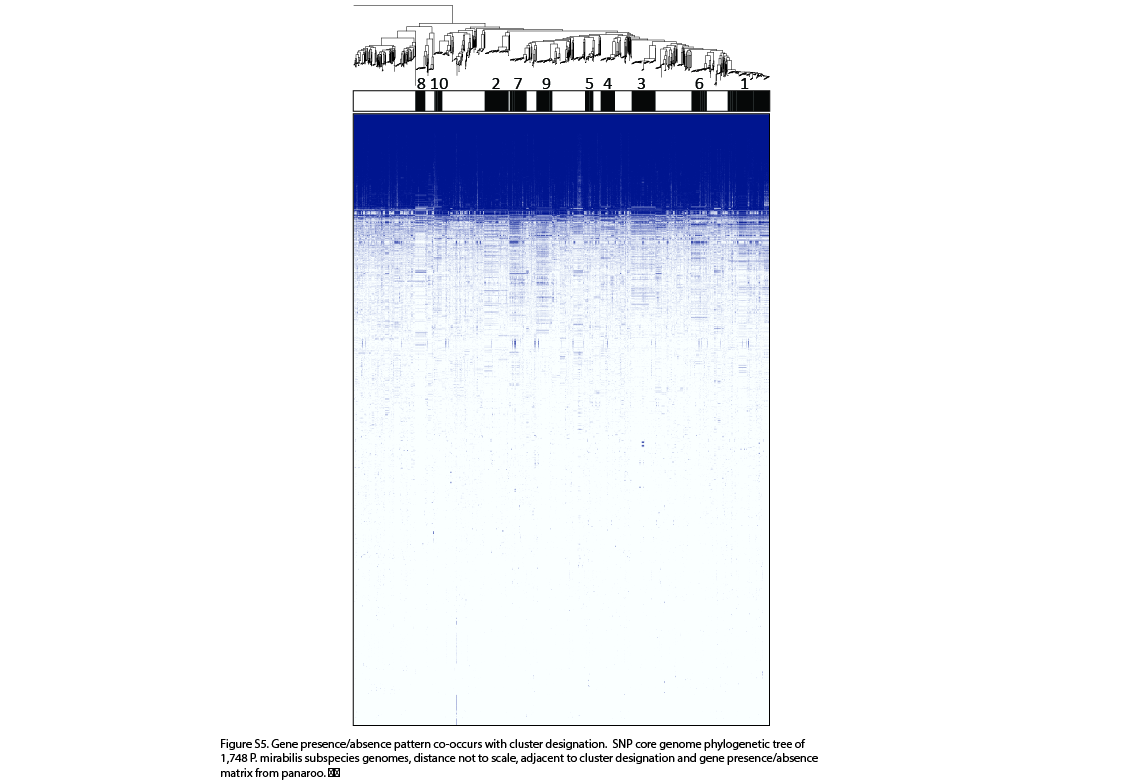

Supplement: Figure S5 — Core genome phylogenetic tree and gene presence/absence matrix. [file msystems.00159-23-s0005.tif]
