# Supplementary material for: Uncharacterized and lineage-specific accessory genes within the Proteus mirabilis pan-genome landscape
Source: mSystems. 2023 Jun 21;8(4):e00159-23. doi: 10.1128/msystems.00159-23 (PMC10469602; doi:10.1128/msystems.00159-23)
Supplement: Document S1 — Commands used in this study. [file msystems.00159-23-s0006.docx]

**Document S1.** Commands used in this study

**FastANI**

fastANI --ql /scratch/gdlab/rfpotter/Proteus/fastani_in/genomepaths.txt --rl /scratch/gdlab/rfpotter/Proteus/fastani_in/genomepaths1.txt -o /scratch/gdlab/rfpotter/Proteus/fastani_out_PM_only/fastani_out.txt -t ${SLURM_CPUS_PER_TASK} –matrix

**QUAST**

quast.py /scratch/gdlab/rfpotter/Proteus/fasta/${ID}.fna -l ${ID} -o /scratch/gdlab/rfpotter/Proteus/quast/${ID}

**Prokka**

prokka /scratch/gdlab/rfpotter/Proteus/${ID}.fasta --outdir /scratch/gdlab/rfpotter/Proteus/prokka/${ID} 1 --locustag ${ID} --mincontiglen 500 --gram neg --prefix ${ID} --force --notrna --norrna

**Panaroo**

/opt/apps/labs/gdlab/software/env/bin/panaroo -i /scratch/gdlab/rfpotter/Proteus /*.gff -o /scratch/gdlab/rfpotter/Proteus /results --clean-mode moderate -a core --core_threshold .99 -t 12 --search_radius 10 --refind_prop_match 100

**SNP-sites**

snp-sites -mv -o /scratch/gdlab/rfpotter/Proteus/panaroo_one/results/aligned_gene_snp/${ID} /scratch/gdlab/rfpotter/Proteus/panaroo_one/results/aligned_gene_sequences/${ID}.aln.fas

**FastTree**

FastTree -nt -gtr -gamma /scratch/gdlab/rfpotter/Proteus/panaroo_one/results/snp.snp_sites.aln > /scratch/gdlab/rfpotter/Proteus/panaroo_one/results/fasttree.nwk

**Scoary**

/opt/apps/scoary/1.6.16/bin/scoary -g /scratch/gdlab/rfpotter/Proteus/panaroo_one/results/gene_presence_absence.Rtab -t /scratch/gdlab/rfpotter/Proteus/panaroo_one/results/biggroup.txt -o /scratch/gdlab/rfpotter/Proteus/panaroo_one/results/biggroup

**RAxML**

raxmlHPC-PTHREADS -s ${indir}/core_genome_snp.snp_sites.aln -w ${outdir}  -n core  -m GTRGAMMA -f a   -T ${SLURM_CPUS_PER_TASK} -N 100-p 12345 -x 54321

**ANI Heatmap**

install.packages("reshape2")

install.packages("gplots")

library("reshape2")

library("gplots")

### get data, convert to matrix

x <- read.table("C:\\PROTEUS_MASTER\\ani\\fastani_out2.txt")

matrix <- acast(x, V1~V2, value.var="V3")

matrix[is.na(matrix)] <- 70

PMlist <- read.csv("C:/PROTEUS_MASTER/ani/PMlist.csv", header=FALSE)

matrix2 <- matrix[!rownames(matrix) %in% PMlist, ]

matrix3 <- matrix2[ , colnames(matrix2) %in% PMlist]

### define the colors within 2 zones

breaks = seq(min(matrix3), max(100), length.out=100)

gradient1 = colorpanel( sum( breaks[-1]<=98 ), "red", "white" )

gradient2 = colorpanel( sum( breaks[-1]>98 & breaks[-1]<=100), "white", "blue" )

hm.colors = c(gradient1, gradient2)

heatmap.2(matrix3, scale = "none", trace = "none", col = hm.colors, cexRow=.30, cexCol=.30)

pdf(file="C:\\PROTEUS_MASTER\\ani\\test.pdf)

heatmap.2(matrix3, scale = "none", trace = "none", col = hm.colors, cexRow=.30, cexCol=.30)

dev.off()

**tSNE**

if (!requireNamespace("BiocManager", quietly = TRUE))

install.packages("BiocManager")

BiocManager::install("Rtsne")

library(vegan)

library('Rtsne')

library(ggplot2)

train<- read.csv("C:\\PROTEUS_MASTER\\tsne\\matrix_subspecies1.csv")

Labels<-train$Group

train$Group<-as.factor(train$Group)

set.seed(42)

tsne <- Rtsne(train[,-1], dims = 2, perplexity=40, verbose=TRUE, max_iter = 5000, check_duplicates = FALSE)

tsne_plot <- data.frame(x = tsne$Y[,1], y = tsne$Y[,2], col = train$Group)

ggplot(tsne_plot) + xlab ("Dimension 1") + ylab("Dimension 2") + geom_point(aes(x=x, y=y, color=col)) + scale_color_manual(breaks = c("Subspecies 1 (Group 1)", "Subspecies 1 (Group 2)", "Subspecies 1 (Group 3)","Subspecies 2", "Subspecies 3", "Not grouped" ), values=c('#e41e1b', '#984ea3', '#4caf4a', '#377eb8', '#ff8000', '#696969'))

ggplot(tsne_plot) + xlab ("Dimension 1") + ylab("Dimension 2") + geom_point(aes(x=x, y=y, color=col)) + scale_color_manual(breaks = c("Cluster1", "Cluster2", "Cluster3","Cluster4", "Cluster5", "cluster6", "cluster7", "cluster8","cluster9", "cluster10", "Not.Cluster" ), values=c('#e41e1b', '#984ea3', '#4caf4a', '#377eb8', '#ff8000', '#7180B9', '#FFC0CB', '#1B998B', '#5B2333', '#00fa9a', '#696969'))

**Gene Prescence Absence and Newick Tree**

%matplotlib inline

import matplotlib.pyplot as plt

import seaborn as sns

sns.set_style('white')

import os

import pandas as pd

import numpy as np

from Bio import Phylo

t = Phylo.read('subspecies1_newick.txt', 'newick')

t.root_with_outgroup("SRR11103428")

mdist = max([t.distance(t.root, x) for x in t.get_terminals()])

roary = pd.read_table('gene_presence_absence_roary.csv',

sep=',',

low_memory=False)

# Set index (group name)

roary.set_index('Gene', inplace=True)

# Drop the other info columns

roary.drop(list(roary.columns[:13]), axis=1, inplace=True)

roary.replace('.{2,100}', 1, regex=True, inplace=True)

roary.replace(np.nan, 0, regex=True, inplace=True)

idx = roary.sum(axis=1).sort_values(ascending=False).index

roary_sorted = roary.loc[idx]

roary_sorted = roary_sorted[[x.name for x in t.get_terminals()]]

roary2 = pd.read_table('cluster_binary.txt',sep='\t', low_memory=False)

roary2 = roary2[[x.name for x in t.get_terminals()]]

with sns.axes_style('whitegrid'):

fig = plt.figure(figsize=(17, 10))

ax1=plt.subplot2grid((1,40), (0, 10), colspan=30)

a=ax1.imshow(roary_sorted.T,cmap=plt.cm.Blues,

vmin=0, vmax=1,

aspect='auto',

interpolation='none',

)

ax1.set_yticks([])

ax1.set_xticks([])

ax1.axis('off')

ax = fig.add_subplot(1,2,1)

ax=plt.subplot2grid((1,40), (0, 0), colspan=10, facecolor='white')

fig.subplots_adjust(wspace=0, hspace=0)

ax1.set_title('Roary matrix\n(%d gene clusters)'%roary.shape[0])

Phylo.draw(t, axes=ax,

show_confidence=False,

label_func=lambda x: None,

xticks=([],), yticks=([],),

ylabel=('',), xlabel=('',),

xlim=(-0.01,mdist+0.01),

axis=('on',),

title=('parSNP tree\n(%d strains)'%roary.shape[1],), do_show=False

)

plt.savefig('tree_PA.eps')
